# The Relationship between Gray Matter Quantitative MRI and Disability in Secondary Progressive Multiple Sclerosis

**DOI:** 10.1371/journal.pone.0161036

**Published:** 2016-08-11

**Authors:** René-Maxime Gracien, Alina Jurcoane, Marlies Wagner, Sarah C. Reitz, Christoph Mayer, Steffen Volz, Stephanie-Michelle Hof, Vinzenz Fleischer, Amgad Droby, Helmuth Steinmetz, Frauke Zipp, Elke Hattingen, Ralf Deichmann, Johannes C. Klein

**Affiliations:** 1 Department of Neurology, Goethe University, Frankfurt/Main, Germany; 2 Department of Neuroradiology, Goethe University, Frankfurt/Main, Germany; 3 Brain Imaging Center, Goethe University, Frankfurt/Main, Germany; 4 Department of Neurology, Johannes Gutenberg University, Mainz, Germany; 5 Neuroimaging Center (NIC) of the Focus Program Translational Neuroscience (FTN), Johannes Gutenberg-University, Mainz, Germany; 6 Nuffield Department of Clinical Neurosciences, University of Oxford, Oxford, United Kingdom; Linköping University, SWEDEN

## Abstract

**Purpose:**

In secondary progressive Multiple Sclerosis (SPMS), global neurodegeneration as a driver of disability gains importance in comparison to focal inflammatory processes. However, clinical MRI does not visualize changes of tissue composition outside MS lesions. This quantitative MRI (qMRI) study investigated cortical and deep gray matter (GM) proton density (PD) values and T1 relaxation times to explore their potential to assess neuronal damage and its relationship to clinical disability in SPMS.

**Materials and Methods:**

11 SPMS patients underwent quantitative T1 and PD mapping. Parameter values across the cerebral cortex and deep GM structures were compared with 11 healthy controls, and correlation with disability was investigated for regions exhibiting significant group differences.

**Results:**

PD was increased in the whole GM, cerebral cortex, thalamus, putamen and pallidum. PD correlated with disability in the whole GM, cerebral cortex, putamen and pallidum. T1 relaxation time was prolonged and correlated with disability in the whole GM and cerebral cortex.

**Conclusion:**

Our study suggests that the qMRI parameters GM PD (which likely indicates replacement of neural tissue with water) and cortical T1 (which reflects cortical damage including and beyond increased water content) are promising qMRI candidates for the assessment of disease status, and are related to disability in SPMS.

## Introduction

MS is an immune-mediated chronic disease of the CNS. The diagnosis and treatment of MS is based on clinical observations and supported by MRI [1]. As WM lesions are the most prominent MS-related pathology, MS was initially considered to be primarily a WM disease. However, more recent histopathological and MRI findings have demonstrated extensive gray matter (GM) involvement in MS [2]. In routine clinical practice, conventional MRI contrasts are the basis for diagnosis and evaluating lesion load, also identifying new lesions in longitudinal investigations [3]. Unfortunately, the association between clinical disability and conventional radiological findings is poor [4].

The transition to secondary progression is important clinically, because therapy options are limited for this phase. Importantly, the transition to Secondary Progressive Multiple Sclerosis (SPMS) can be delayed by disease modifying therapy [5]. In SPMS, diffuse inflammation accompanied by a widespread neurodegenerative component resulting in global axonal loss seems to become more prominent than in relapsing-remitting MS (RRMS), and is thought to be the primary cause of disability progression in SPMS [6–8]. At this stage of the disease, an intact blood brain barrier is thought to compartmentalize inflammation within the CNS, which might explain the low effectiveness of most of the current therapies [8]. Diffuse microstructural changes in GM, caused by global inflammation and neurodegeneration, are not visible on routine clinical MRI. Therefore, as opposed to relapsing-remitting MS, the monitoring of SPMS patients is primarily based on clinical measurements. Changes of tissue composition in GM, where this pathology actually takes place, cannot be assessed directly with conventional MRI.

Quantitative MRI (qMRI) techniques allow for the direct measurement of tissue parameters in vivo, providing a window into the microstructural tissue composition and its changes in MS. qMRI detects pathological processes in tissue that appears normal on conventional MR images, and the measured tissue parameters have been shown to correlate with disability in RRMS and mixed collectives:

Prolongation of the T1 relaxation time in normal appearing brain tissues has been shown for various collectives of MS patients, including primary-progressive MS patients [9,10], SPMS patients [9,11] and RRMS patients [12–14]. Parry et al. demonstrated a correlation of T1 relaxation times in normal appearing white matter and MS lesions with disability in a mixed collective of RRMS and SPMS patients [11]. Normal appearing white matter T1 values correlated with disability in a collective of primary-progressive MS patients [10]. Furthermore, cortical T1 histogram mode was shown to be associated with clinical disability in a mixed RRMS and SPMS group [9], and whole-brain T1 correlated with results of the nine-hole peg test [15], an index of arm function, but not the Expanded Disability Status Scale (EDSS), a multimodal, integrative index of global disability, in a group of early RRMS patients [13].

In RRMS patients, the proton density (PD) in the frontal white matter inside and adjacent to the corpus callosum was found to correlate strongly with EDSS [16].

In summary, a correlation between T1 values measured in various regions and the degree of disability has been shown in mixed collectives including RRMS and SPMS patients, but it is unclear whether these results are representative for SPMS patients, or whether the correlation with disability was driven by the combination of patients at early and late stages of disease. For the parameter PD, which indicates replacement of neural tissue by water in the cortex, no data specific to SPMS patients are available.

There is an as yet unmet need for better imaging surrogates of disease state in SPMS, where information from conventional MRI does not sufficiently reflect the ongoing pathology. In this study, we explore qMRI measurements taken from cortical and subcortical GM regions of interest (ROIs) in SPMS patients. We assess parameter changes relative to a collective of healthy subjects, and explore the relationship between qMRI tissue parameters and clinical disability in SPMS. The hope is to provide clinicians and researchers with a means to measure neurodegeneration directly, and to provide a suitable and clinically relevant imaging surrogate of neuronal damage in SPMS.

The study presented here aims at an assessment of microstructural changes in cerebral tissue apart from MS lesions. T1 and PD values in the cerebral cortex and deep GM of SPMS patients were systematically investigated with advanced qMRI and segmentation methods, comparing the results with values measured on a matched collective of healthy controls and evaluating the correlation with the EDSS.

## Materials and Methods

### Participants

11 patients (4 male) with SPMS and 11 age-matched healthy control subjects (5 male) participated in the study. Healthy controls were free from neurological or psychiatric disease, as assessed by systematic history evaluation and clinical examination. Further exclusion criteria were: brain surgery, drug abuse, diabetes mellitus and severe arterial hypertension. Data from an overlapping collective have been reported earlier, evaluating the predictive value of qMRI regarding gadolinium enhancing lesions [17]. Patients were scored on the EDSS by an MS specialist [18]. Written informed consent was given, and the study was approved by the ethics committee of the State Medical Association of Rhineland-Palatine and by the Institutional Review Board. The study was conducted according to the principles expressed in the Declaration of Helsinki.

### Data acquisition

Measurements were performed on a 3 Tesla whole body MR scanner (Trio, Siemens Medical Solutions, Erlangen, Germany; radiofrequency transmission: body coil, radiofrequency reception: 8-channel phased-array head coil).

The variable flip angle method was applied for T1 and PD mapping [19]. This method is based on two spoiled 3D gradient echo acquisitions at different excitation angles, resulting in a PD-weighted and a T1-weighted data set. A FLASH-EPI hybrid readout was applied [20] to increase the signal-to-noise ratio. The parameters were: isotropic spatial resolution of 1mm, field-of-view 256 x 224 x 160 mm^3^, TR/TE/α1/α2 = 16.4 ms/6.7 ms/4°/24°, BW = 222 Hz/Pixel. The total scan duration for both data sets was 9:48 min.

Non-uniformities of the transmitted radiofrequency field (B1) were measured as described before [21]: two multi-slice gradient echo data sets are recorded, one after full spin relaxation and one after radiofrequency irradiation which rotates the longitudinal magnetization by a certain angle. As the quotient of both data sets yields the cosine of this angle, B1 can be obtained by comparing the local angle with the nominal value which was set to 45°. The parameters were: isotropic spatial resolution: 4 mm, field-of-view as above, TR/TE/α = 11 ms/5 ms/11°, BW = 260 Hz/Pixel. The duration of B1 mapping was 0:53 min.

For the evaluation of residual signal losses induced by T2* relaxation effects during the finite TE of 6.7 ms, two multi-slice gradient echo data sets with different TE were recorded with the following parameters: isotropic spatial resolution: 2 mm, field-of-view as above, TR/TE1/TE2/α = 1336 ms/4.3 ms/11 ms/50°, BW = 292 Hz/Pixel. The scan duration for both data sets was 5 min.

### Data processing and analysis

Data processing was performed with custom-built functions written for Matlab (MathWorks, Natick, MA) and shell scripts using tools from the FMRIB Software Library [22].

B1 was obtained as described above and in the literature [21]. Quantitative T1-maps were calculated from the variable flip angle data [19]. In detail, S_i_/tan(α_i_) was plotted versus S_i_/sin(α_i_) where S_1/2_ are the local signal amplitudes in the PD and T1 weighted data sets, respectively, and α_1/2_ are the respective excitation angles, corrected for B1 inhomogeneities. Additionally, data were corrected for the effect of insufficient spoiling of transverse magnetization [23]. PD maps were derived as reported before [24]: in summary, the PD weighted data set was corrected for any T1, T2* and B1 bias and non-uniformities of the receive coil sensitivity profile [24]. PD values were normalized by defining a value of 100% in CSF.

Synthetic T1-weighted anatomical MP-RAGE data sets were calculated from the quantitative T1 and PD maps, following the procedure described in [25]. The calculation was performed for the following parameters: isotropic spatial resolution: 1 mm, field-of-view as above, TR/TI/α = 1900 ms/900 ms/9°. Since the input maps were corrected for T2* effects, the nominal TE value of the synthetic MP-RAGE data is zero.

After skull-stripping the data sets using the FMRIB Software Library tool “BET” for every participant, the software “FAST” [26] was applied and the anatomical MP-RAGE data set was segmented into GM, WM and CSF partial volume estimate (PVE) maps. The calculated GM PVE map was used to create a cortex PVE map, eliminating all voxels with a PVE < 0.95 and voxels belonging to non-cortical structures. The latter was achieved by eliminating all voxels that were part of an exclusion mask, comprising masks of deep GM (calculated with “FIRST”), masks of the white matter with filled ‘holes’ to eliminate lesions (obtained with “FAST”) and masks of the cerebellum and the ventricles. For removal of artefacts and non-cortical tissue from the cortex PVE map, a threshold of absolute T1 between 1200 and 1600ms was applied to the final map [24,27]. Lesions still remaining in the cortical PVE maps were manually removed as described in detail below. The value of this cortical segmentation method has been demonstrated earlier for cohorts of RRMS patients [28–30]. The final cortical PVE maps were intersected with the T1 and PD maps, and cortical mean values of these parameters were determined by calculating weighted averages of the individual maps using the local values of the cortical PVE maps as weighting factors.

Additionally, for every single participant, a segmentation of the deep GM structures, which are anatomically best suited for an accurate segmentation (thalamus, caudate nucleus, putamen and globus pallidus), was generated with “FIRST” [31]. To avoid partial voluming with CSF, the outermost voxels of the respective ROI were removed by morphological erosion with a 3-dimensional 3x3x3 mm^3^ kernel. The respective deep GM structures of both hemispheres were combined to yield one ROI per structure. Additionally, a global GM map was created from the deep GM and cortex maps. After removal of lesions as described below, the respective maps were applied to the T1 and PD maps and mean values for T1 and PD across each ROI were calculated. The study focused on GM changes, as GM pathology is not well respresented by clinical MRI. Still, in order to also investigate normal appearing WM (NAWM) separately, WM masks were obtained with ‘FAST’ from the FSL toolbox and eroded with a 6x6x6 mm^3^ kernel in order to reduce partial volume effects. After manual removal of any remaining MS lesions (see below), the masks were applied to the T1 and PD maps and the mean T1 and PD values in NAWM were extracted.

In detail, the manual removal of lesions still remaining in the GM and WM ROIs was performed as follows: the respective GM and WM ROIs were carefully inspected together with the MP-RAGE datasets by a researcher experienced in the management of patients with MS. If a remaining MS lesion was identified within those ROIs, these were manually edited with ‘FSLVIEW’ and all voxels of the ROI overlapping with the identified MS lesion were removed. S1 Fig demonstrates examples of the final ROIs.

To assess T1 and PD values in the whole brain, voxels containing CSF only needed to be excluded. For this purpose, a threshold was applied to the skull-stripped quantitative maps removing voxels with T1 values above 1600ms [24,27] from the T1 maps, and voxels with PD values above 84.44 pu from the PD maps. The PD value 84.44 pu was chosen as it corresponds to a T1 value 1600ms according to the Fatouros equation [32] with the parameters proposed for 3 Tesla [33]. Afterwards, mean T1 and PD values across the whole brain were calculated.

### Statistics

Statistical analyses were carried out with SPSS for Windows (20.0.0). Non-parametric testing was used (Mann-Whitney-U) for group comparisons of the respective mean parameter values. As evaluation of the relationship between quantitative and clinical parameters is only meaningful for ROIs in which T1 or PD are significantly increased in the MS group as compared to the control group, correlations of T1 and PD with EDSS, disease duration, gender and age were assessed only for the respective regions, using Spearman’s rank correlation coefficient. For all statistical procedures, tests where p was <0.05 were considered significant.

## Results

**Fig 1:**
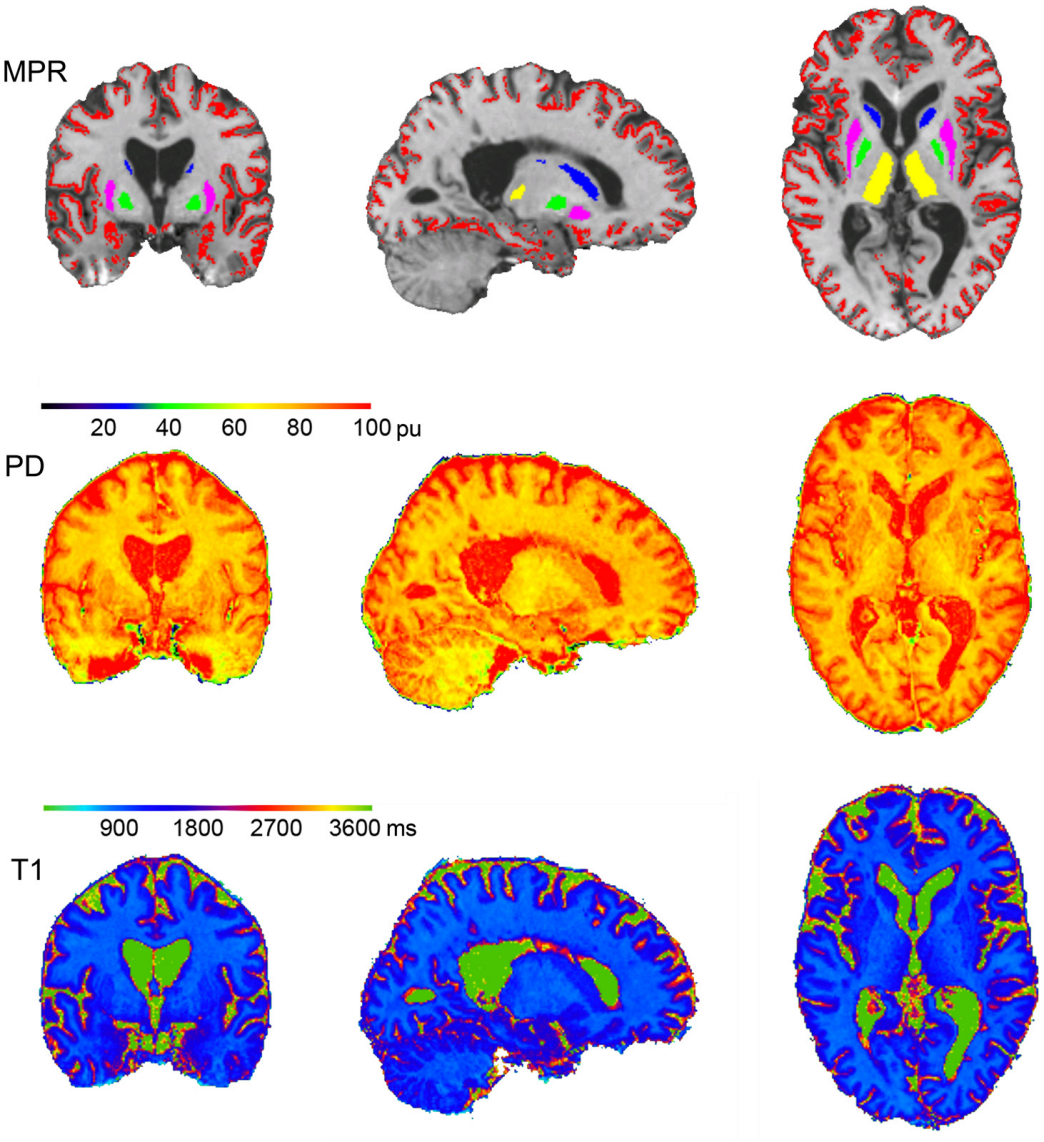
Representative example for calculated quantitative maps and segmentation of the synthetic anatomical MP-RAGE data set. synthetic MP-RAGE data (top) with an overlay showing the identified volumes of the cortex (red) and deep gray matter structures in colors (caudate nucleus in blue, thalamus in yellow, putamen in pink and pallidum in green), the PD map (second row) and T1 map (bottom). All data are shown for three orthogonal slices in MNI 152 standard space (center x = 19.00, y = -2.00, z = 3.00).

Average age, EDSS and disease duration of the 11 patients examined (mean ± standard deviation) were: Age 46.8±11.03 years, EDSS 5.8±1.88, range 3–8.5, disease duration 15.3±6.75 years. The healthy control group did not differ in age (43.6±11.17 years; p = 0.70). Fig 1 shows the calculation and segmentation of the synthetic anatomical MP-RAGE data set and the quantitative maps for a representative subject. The synthetic MP-RAGE is overlaid with the masks used for the cortex and deep GM structures (top), the PD map (second row) and the T1 map (bottom). The data sets are shown for three orthogonal slices in MNI 152 standard space (x = 19.00, y = -2.00, z = 3.00).

Table 1 lists absolute T1 and PD values in the different ROIs (mean ± standard deviation) for the SPMS and control cohorts. PD was significantly increased in the MS group in the whole normal appearing GM (NAGM), cerebral cortex, pallidum, putamen, thalamus, NAWM and the whole brain, but not in the caudate nucleus. T1 increase was significant in the NAGM, cerebral cortex, NAWM and whole brain only. Furthermore, there was a trend for increased T1 in the thalamus.

**Table 1:** Mean T1 relaxation times and proton densities. Data are shown in the different regions for the patients and the healthy controls (mean and standard deviation across group). * indicates significant group comparisons. NAGM = normal appearing gray matter. NAWM = normal appearing white matter.

Parameter	ROI	Patients/HC	Mean	SD	p value
PD	NAGM	Patients	83.6	1.93	0.002*
		HC	80.7	1.60	
	Cortex	Patients	83.8	1.91	0.001*
		HC	80.8	1.67	
	Caudate nucleus	Patients	81.8	2.51	0.151
		HC	80.2	2.01	
	Pallidum	Patients	77.1	2.63	0.013*
		HC	74.4	1.76	
	Putamen	Patients	82.5	2.91	0.023*
		HC	79.7	1.69	
	Thalamus	Patients	78.7	2.01	0.005*
		HC	75.6	2.60	
	NAWM	Patients	71.7	2.49	0.001*
		HC	67.9	1.68	
	Whole	Patients	72.5	1.36	0.008*
	Brain	HC	70.9	0.85	
T1	NAGM	Patients	1415.9	30.25	0.016*
		HC	1390.7	16.79	
	Cortex	Patients	1425.9	27.09	0.019*
		HC	1403.0	15.93	
	Caudate nucleus	Patients	1326.7	48.93	0.652
		HC	1343.1	43.30	
	Pallidum	Patients	1003.4	49.08	0.478
		HC	1016.2	42.15	
	Putamen	Patients	1264.5	54.64	0.401
		HC	1278.6	39.96	
	Thalamus	Patients	1252.6	83.98	0.065
		HC	1184.4	54.55	
	NAWM	Patients	911.0	41.16	0.005*
		HC	865.8	28.75	
	Whole	Patients	1161.6	33.79	0.047*
	Brain	HC	1134.4	16.65	

There was no significant correlation between the tissue parameters and age, gender, or disease duration for any ROI.

Table 2 lists the correlation coefficients between EDSS and the tissue parameters T1 and PD for regions in which T1 or PD are significantly increased in the MS group. EDSS was correlated with PD values for the whole NAGM, cerebral cortex, pallidum and putamen, but not for the thalamus, NAWM or whole brain tissue. Correlations between EDSS and T1 were found for the whole NAGM and cerebral cortex. Furthermore, a trend could be observed for the whole brain. Neither GM T1 (r = 0.13, p = 0.70) nor PD (r = 0.17, p = 0.62) values were correlated with the disease duration.

**Table 2:** Spearman’s rank correlation coefficients for correlations between EDSS score and the tissue parameters T1 and PD for regions in which T1 or PD are significantly increased in the MS group. * indicates significant correlations. NAGM = normal appearing gray matter. NAWM = normal appearing white matter.

ROI			PD	T1
NAGM	EDSS	correlaton coefficient r	0.629*	0.779*
		p value	**0.038**	**0.005**
Cortex	EDSS	correlaton coefficient r	0.606	0.779
		p value	0.048*	0.005*
Pallidum	EDSS	correlaton coefficient r	0.661	
		p value	0.027*	
Putamen	EDSS	correlaton coefficient r	0.670	
		p value	0.024*	
Thalamus	EDSS	correlaton coefficient r	0.401	
		p value	0.222	
NAWM	EDSS	correlaton coefficient r	0.469	0.383
		p value	0.145	0.245
Whole	EDSS	correlaton coefficient r	0.205	0.588
brain		p value	0.545	0.057

**Fig 2:**
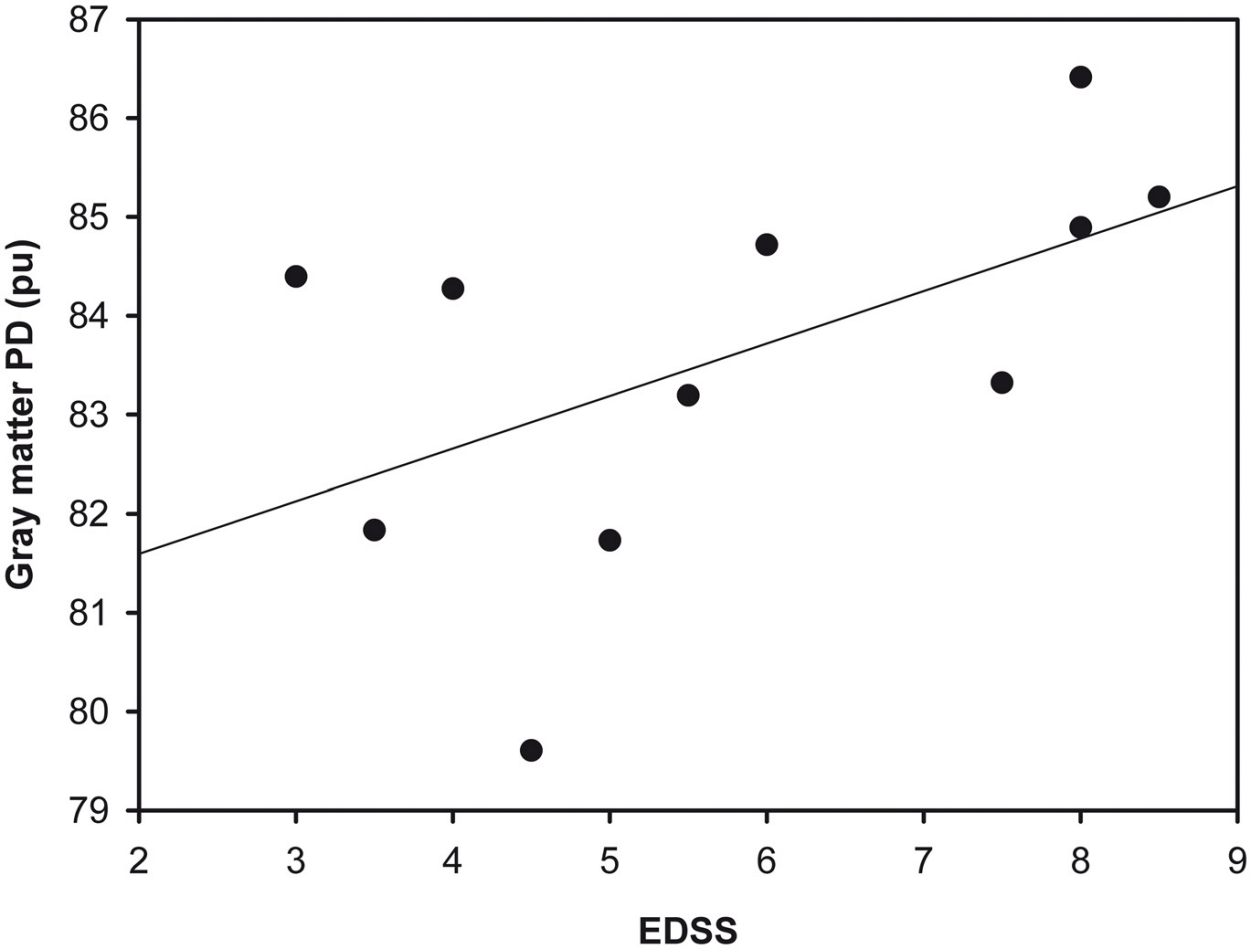
Scatter plot showing EDSS and PD values in the gray matter.

**Fig 3:**
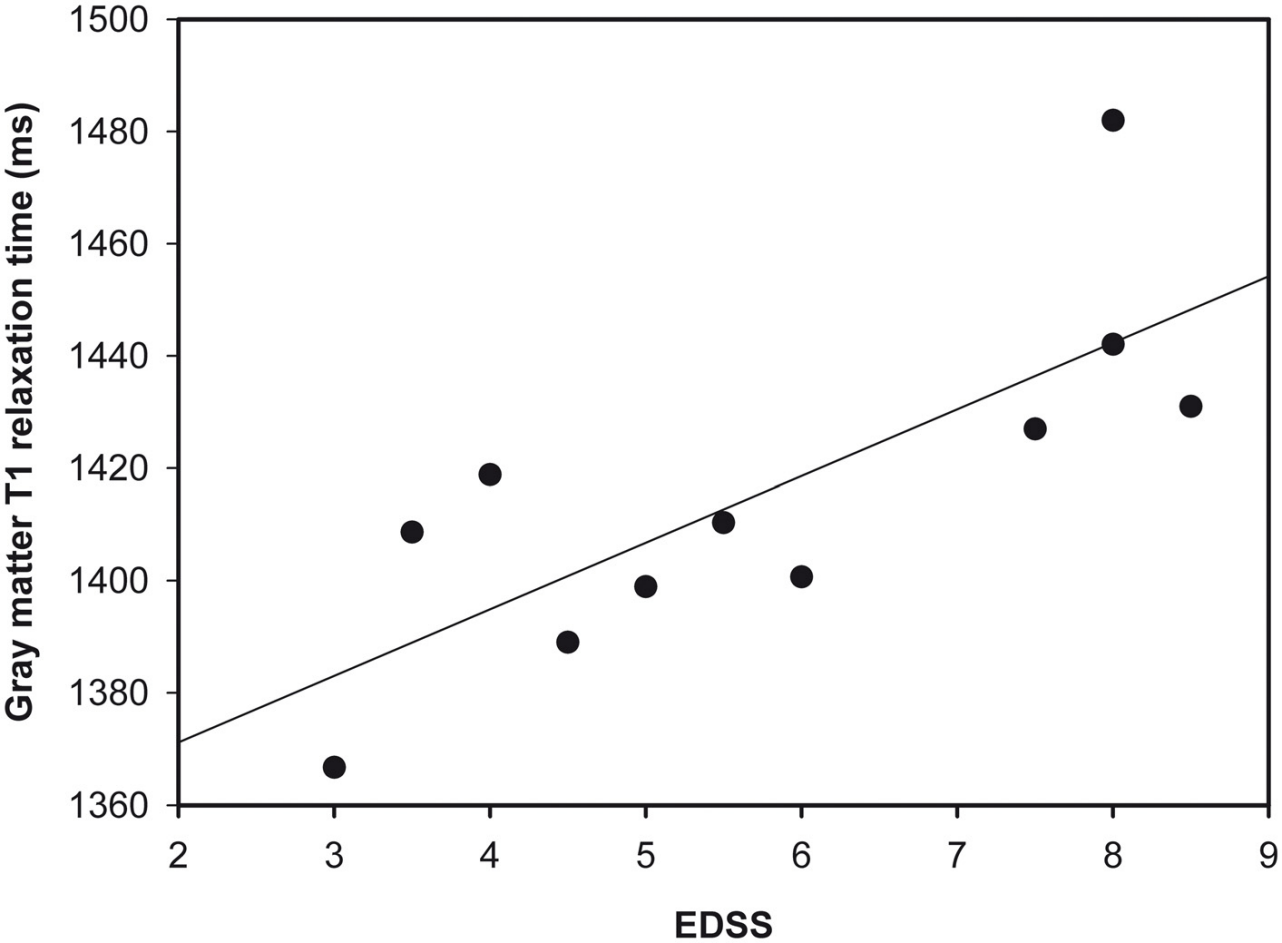
Scatter plot showing EDSS and gray matter T1 values.

For the whole NAGM, Figs 2 and 3 show the relationship between EDSS and PD (Fig 2), and between EDSS and T1 (Fig 3).

## Discussion

This study investigates cortical and deep GM damage in SPMS patients using qMRI measurements of absolute PD and T1 values as potential imaging surrogates of disease state and disability. When MS patients enter the secondary chronic progressive phase, neurodegeneration, rather than localized inflammatory disease activity, appears to drive progression of clinical disability [6,7]. Diffuse neuronal damage, unlike the localized loss of myelin and axons produced in focal lesions, is understood to play an important role in the accumulation of disability [34].

So far, radiological assessment in SPMS patients relies on conventional MRI, usually by comparing the lesion load with previous scans and visually estimating the global brain atrophy. However, it is the tissue outside of these lesions where the pathogenic processes mainly occur in SPMS–indeed, disability is driven by damage to tissue outside overt lesions that remains inaccessible to routine MRI assessment [34]. Clearly, there is a need for new imaging surrogates of diffuse neuronal damage in SPMS. qMRI offers a window of insight into such microstructural changes.

Our data show that PD in the cerebral cortex, thalamus, putamen and pallidum is increased in our cohort of SPMS patients. Previous histopathological studies have identified neurodegeneration and subsequent loss of neurons as defining pathological processes in SPMS. The quantitative parameter PD assesses the tissue water content. For SPMS patients, PD might be sensitive to replacement of neural tissue by interstitial water, thus being sensitive to neuronal loss, but also to loss of supporting glial structures or myelin, or cell swelling. Gross edema is unlikely to occur in SPMS, but could form an alternative explanation for increased PD in other conditions. Attractively, our observations suggest that PD may be a suitable parameter to assess neurodegenerative changes in SPMS.

T1 measurements in the cortex inform about the actual composition of tissue, including, but also reaching beyond water content. T1 reflects a complex interplay of factors such as myelin content, iron deposits, axonal density and cellularity. T1 changes in MS indicate loss of myelin, neuronal/axonal loss and gliosis [35,36]. Cortical and whole GM T1 relaxation time was prolonged in our SPMS group, and there was a trend for T1 prolongation in the thalamus. Previous investigations [9,11] have reported similar results, demonstrating T1 increase for the cortex and thalamus but not for other deep GM structures in SPMS. It is quite possible that power issues prevented detection of thalamic T1 changes in our study (p = 0.065), owing to the relatively small group size.

While both T1 and PD changes were observed in the cerebral cortex, the question arises why PD only was increased in three out of four deep GM regions studied (Table 1). A common source of error in T1 quantification via the variable flip angle method is a lack of accuracy in the underlying B1 maps. In fact, an error of only 5% in B1 would yield a 10% deviation between real and apparent T1 values. B1 errors may occur in the vicinity of bones and fat due to susceptibility and chemical shift effects, respectively. Furthermore, longitudinal relaxation effects may yield errors inside or close to CSF, due to long T1 values. However, it is unlikely that any of these effects played a major role for the data acquired here: the B1 mapping method employs a very short TE of 5ms and is based on the quotient of two data sets, so signal losses due to susceptibility and chemical shift effects would have rather minor effects. In addition, data are carefully corrected for longitudinal relaxation effects in tissue and CSF, applying the approximate respective T1 values [21]. Furthermore, it should be taken into account that PD maps are derived from PD-weighted data sets via correction for T1 and B1 effects, such that an error in B1 and therefore in T1 would also yield erroneous PD values.

A nother factor for the differences observed for T1 and PD increases might be that, even though T1 values are affected by the water content, other processes driving T1 changes in SPMS, such as gliosis, myelin loss and reduced axonal density, play a more prominent role in the cerebral cortex than in the deep gray. Moreover, an increased deposition of iron in the deep GM [37] could shorten T1 [38], and counteract the T1 prolonging effects of the processes listed above, thus producing contrasting observations in the cortical and deep GM.

In contrast to the situation with T1, increased water content in tissue, as reflected by increased PD, appears to be widespread in both the cortex and deep GM ROIs. While it is not possible to isolate a single cause of the T1 changes from our data, they suggest that the pathological processes in cortical and deep grey are not identical. An alternative explanation would be a higher sensitivity of PD in the detection of neurodegeneration in SPMS.

To investigate the clinical relevance of our findings, we performed correlation analysis between the qMRI measurements in the GM regions identified above and EDSS, a score for the assessment of disability in MS (Table 2). EDSS was correlated with whole NAGM PD, cortical PD and the PD in two out of the three deep GM structures that showed significant differences between the groups. In conclusion, the quantitative tissue parameter PD in GM was closely related to disability in our SPMS cohort while sampling from tissue outside of lesions, i.e. in the areas where neurodegeneration primarily takes place.

In the study presented here, a significant correlation with the EDSS could also be shown for cortical and whole NAGM T1. These results are congruent to a previous study showing cortical T1 to be correlated with disability in a mixed RRMS and SPMS group [9]. Interestingly, GM T1 and PD values were not correlated with the disease duration. This result might be explained by the fact that patients with a less aggressive course of disease would accumulate a lower degree of disability and tissue damage even after a long disease duration.

Naturally, patients with higher EDSS scores tend to be older than patients with lower EDSS scores. However, as GM T1 and PD values decrease with age [39,40], this age-dependence would not explain the positive correlation between EDSS scores and T1 or PD values.

Interestingly, T1 and PD values were increased in NAWM, but a relationship with the degree of disability could only be observed for the respective values in NAGM. In NAWM, no such relationship was found, which indicates that GM might be a more relevant target for the assessment of global neurodegenerative processes with qMRI techniques in SPMS.

We also found T1 and PD increases across the whole brain. These, however, would strongly depend on the macroscopic lesion load. No correlation of T1 and PD values with the EDSS could be observed for the whole brain. These results suggest that the analysis of clearly defined anatomical regions with similar microstructural properties outside of MS lesions is of advantage for the assessment of neurodegeneration in relation to the clinical status in SPMS.

While the present study focuses on GM pathology, previous work performed a more complex WM analysis subdividing the WM outside of MS lesions into NAWM and diffusely abnormal white matter, which seem to reflect independent pathological entities in MS [41].

Our study has some methodological innovations that help avoid partial volume effects and missegmentation, especially in the thin cortical ribbon. Segmentation and ROI definition was performed on synthetic MP-RAGE data sets directly calculated from the T1 and PD maps. Therefore, the quantitative maps share the coordinate space with the anatomical data set, and no registration was required. This reduces the risk of misregistration, partial volume effects, and also of misclassification due to applying segmentation steps in different reference frames. Furthermore, artifacts and misclassified voxels were carefully removed, which is of special importance for the cortical volume which is close to CSF (cf. methods). The removal of non-cortical structures was performed mostly automatically, which is required to pave the way for quantitative T1 and PD mapping towards a potential clinical application.

In contrast to magnetization transfer ratio [42], the tissue parameters T1 and PD can be sampled independent of sequence and hardware parameters with some effort, and the results of an individual patient or of patient groups are then comparable across different sites.

Conventional MRI is widely available and represents standard in clinical routine. Clinical studies might benefit from including qMRI protocols to assess properties of normal appearing tissues, opening a separate window for assessment of the disease. One benefit of the qMRI technique is the ability to provide synthetic anatomies from the data, such as performed in the study presented here. This approach reduces the total MR examination time [43], because conventional MRI data do not need to be acquired separately. Another advantage is the improved contrast that can be obtained by selecting appropriate parameters when calculating these images, and the lack of coil sensitivity artifacts [25]. One disadvantage is that qMRI protocols approved for clinical use are currently only implemented on select commercial MRI scanners. In addition, a widespread application of qMRI techniques in MS is currently difficult as the software packages available from the MRI manufacturers are not optimized for neurological applications. In the light of increased interest in qMRI, as evidenced by our and other groups’ studies [9,11], and existing packages for orthopaedic applications, we hope that qMRI will become more readily available outside of a research environment.

Our study is not without limitations. The number of patients examined is small and the cohort examined might therefore not be representative for SPMS patients in general. Nevertheless, correlations of PD and T1 relaxation time with disability measurements were present in our study group. This fact is encouraging, as the use of MRI surrogates which become significant only in a very large collective would have been more limited. Furthermore, increased partial volume effects in the SPMS group caused by atrophy can never be fully excluded when analyzing the thin cortical ribbon, which might have contributed to the cortical T1 and PD increase. However, this argument would not apply for the deep GM ROIs, where partial volume effects were avoided by performing morphological erosion.

Identification of lesions for exclusion from the ROIs was based on routine MR images, but did not involve special sequences to facilitate the detection of cortical lesions, such as double inversion recovery. However, even DIR detects only 37% of the GM lesions identified by histopathology [44]. Accordingly, the analysis of “normal appearing” tissue does not imply that these tissues are free of lesions. In fact, diffuse small lesions undetectable by MRI are an important pathological hallmark of GM damage in MS [45,46] and would also have contributed to the results in the presented study.

While the degree of disability was quantified with the EDSS in our work, future studies might benefit from more detailed neuropsychological data and other clinical scores such as the Multiple Sclerosis Functional Composite Measure (MSFC) when assessing the relationship of behavioural measures to GM T1 and PD changes in SPMS. Moreover, imaging at higher field strengths might even allow a discrimination of different cortical layers, and their discrete analysis in SPMS.

The study presented here focuses on T1 relaxometry and PD mapping. Future studies may also benefit from incorporating the parameter T2, which has been shown to decrease in the deep GM of MS patients [37], but to increase in NAWM [47] and NAGM [30], potentially reflecting the complex interplay of subcortical iron deposition, increased water content and demyelination.

In conclusion, this study assessed the parameters PD, a potential indicator of replacement of tissue by water, and T1, an indicator of compositional changes including but also beyond increased water content such as gliosis, demyelination and axonal loss. Our results suggest that, cortical and deep GM PD values and cortical T1 values provide biologically meaningful insights into disease status in SPMS. Quantitative T1 and PD mapping helps elucidate pathological processes in SPMS beyond the lesion load, and could potentially allow for the measurement of ongoing pathology in SPMS.

In the future, we hope that clinical studies in SPMS can use qMRI tools to assess ongoing tissue damage, and perhaps even efficacy of treatment as new treatment strategies for SPMS become available. Eventually, qMRI may even help to guide the therapy for individual patients.

## Supporting Information

S1 Fig Examples of final cortical (a) and white matter (b) ROIs. Efforts were taken to exclude lesions (some are marked with red arrows) from the ROIs.
(PDF)

S1 File SPSS data file with extracted T1 and PD values. (SAV)
