# Supplementary figures and images for: The Relationship between Gray Matter Quantitative MRI and Disability in Secondary Progressive Multiple Sclerosis
Source: PLoS One. 2016 Aug 11;11(8):e0161036. doi: 10.1371/journal.pone.0161036 (PMC4981438; doi:10.1371/journal.pone.0161036)

**a**

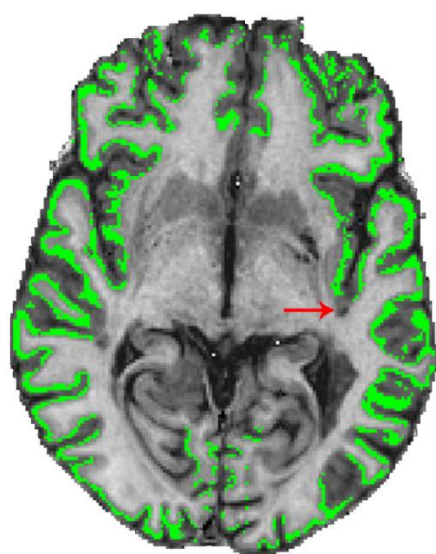

**b**

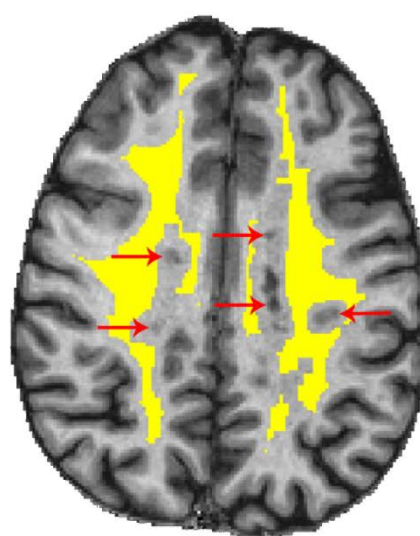

Supplement: S1 Fig — Efforts were taken to exclude lesions (some are marked with red arrows) from the ROIs. (PDF) [file pone.0161036.s001.pdf]
